# CrrAB regulates PagP-mediated glycerophosphoglycerol palmitoylation in the outer membrane of *Klebsiella pneumoniae*

**DOI:** 10.1016/j.jlr.2022.100251

**Published:** 2022-07-13

**Authors:** Lang Sun, Youwen Zhang, Tanxi Cai, Xue Li, Na Li, Zhensheng Xie, Fuquan Yang, Xuefu You

**Affiliations:** 1 Beijing Key Laboratory of Antimicrobial Agents, Institute of Medicinal Biotechnology, Chinese Academy of Medical Sciences & Peking Union Medical College, Beijing, China; 2 Key Laboratory of Protein and Peptide Pharmaceuticals & Laboratory of Proteomics, Institute of Biophysics, Chinese Academy of Sciences, Beijing, China; 3 Key Laboratory of Protein and Peptide Pharmaceuticals & Laboratory of Proteomics, Institute of Biophysics, University of Chinese Academy of Sciences, Beijing, China

**Keywords:** lipidomics, glycerophospholipids (GP), glycerophosphoglycerols (PG), palmitoylation, phospholipids metabolism, proteomics, two-component system CrrAB, palmitoyltransferase PagP, lipopolysaccharide, cationic antimicrobial peptides (CAMP), acyl-PG, acyl-glycerophosphoglycerols, Ara4N, 4-amino-4-deoxy-L-arabinose, CAMP, cationic antimicrobial peptide, CL, cardiolipin, IM, inner membrane, IPA, isopropanol, LPS, lipopolysaccharide, MeOH, methanol, MIC, minimum inhibitory concentration, MRM, multiple reaction monitoring, OM, outer membrane, PE, glycerophosphoethanolamines, PG, glycerophosphoglycerols, GP, glycerophospholipid

## Abstract

The outer membrane (OM) of Gram-negative bacteria is an evolving antibiotic barrier composed of a glycerophospholipid (GP) inner leaflet and a lipopolysaccharide (LPS) outer leaflet. The two-component regulatory system CrrAB has only recently been reported to confer high-level polymyxin resistance and virulence in *Klebsiella pneumoniae*. Mutations in *crrB* have been shown to lead to the modification of the lipid A moiety of LPS through CrrAB activation. However, functions of CrrAB activation in the regulation of other lipids are unclear. Work here demonstrates that CrrAB activation not only stimulates LPS modification but also regulates synthesis of acyl-glycerophosphoglycerols (acyl-PGs), a lipid species with undefined functions and biosynthesis. Among all possible modulators of acyl-PG identified from proteomic data, we found expression of lipid A palmitoyltransferase (PagP) was significantly upregulated in the *crrB* mutant. Furthermore, comparative lipidomics showed that most of the increasing acyl-PG activated by CrrAB was decreased after *pagP* knockout with CRISPR-Cas9. These results suggest that PagP also transfers a palmitate chain from GPs to PGs, generating acyl-PGs. Further investigation revealed that PagP mainly regulates the GP contents within the OM, leading to an increased ratio of acyl-PG to PG species and improving OM hydrophobicity, which may contribute to resistance against certain cationic antimicrobial peptides resistance upon LPS modification. Taken together, this work suggests that CrrAB regulates the palmitoylation of PGs and lipid A within the OM through upregulated PagP, which functions together to form an outer membrane barrier critical for bacterial survival.

## Graphical abstract

**Figure:**
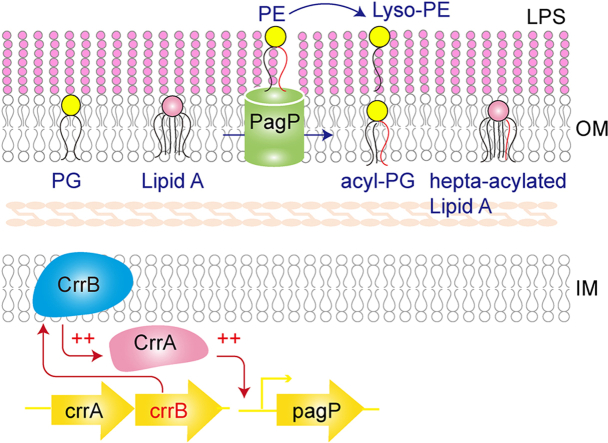

Gram-negative bacteria have a complex cell envelope consisting of an inner membrane (IM), a thin peptidoglycan layer, and an outer membrane (OM) (1). The OM exhibits an asymmetric lipid bilayer with lipopolysaccharide (LPS) in the outer leaflet and phospholipids (GP) in the inner leaflet. The specific structure serves as an effective and selective permeability barrier that protects the cells from toxic agents such as antibiotics, bile salts, and detergents, allowing bacterial survival under adverse conditions (2).

*Klebsiella pneumoniae* is a type of Gram-negative bacteria, which is best known as a pathogen of the human respiratory system that causes pneumonia. The emergence of multidrug resistance especially polymyxin resistance in *K. pneumoniae* necessitates a critical need for new treatments (3, 4). The common mechanisms of polymyxin resistance involve LPS modification, which is mediated by *arnBCADTEF* operon activated by two-component regulatory systems of PhoPQ, PmrAB, CrrAB in *K. pneumoniae* (5, 6). LPS consists of lipid A, core oligosaccharide, and O-specific antigen. Gram-negative bacteria mainly decorate the structure of lipid A, the bioactive component of LPS, in response to polymyxin treatments to promote antimicrobial resistance and interfere with the ability of host recognition. The general lipid A remodeling contains 4-amino-4-deoxy-L-arabinose (L-Ara4N), phosphoethanolamine addition, and palmitoylation, which would lead to perturbation of OM permeability (7). As major constituents of the OM, GP contents may vary with the lipid A alterations to maintain a healthy balance between membrane integrity and lipid A modification. Given that the maintenance of OM is essential for bacterial survival, understanding the GP regulation of OM may empower the development of new therapeutics.

In *Escherichia coli*, the cell membranes are composed of three major GP species including ∼75% glycerophosphoethanolamines (PEs), ∼20% glycerophosphoglycerols (PGs), and 5% cardiolipin (CL) (8, 9). Like *E. coli*, the membrane glycerophospholipids of *K. pneumoniae* mainly consist of PE, PG, and CL. Each phospholipid class comprises a multitude of molecular species defined by acyl chain compositions. GPs are synthesized in the cytoplasm or inner membrane and transported to the OM by specialized transporters. Unlike LPS transport, GP transport between the IM and the OM is bidirectional, which still requires thorough investigation (10). It has been demonstrated that the Mla system and the Tol-Pal complex were important for retrograde transport of GP (11, 12). Recently, AsmA-like proteins YhdP, TamB, and YdbH were reported to be necessary for proper anterograde transport of GP (13, 14).

To prevent uncontrolled breakdown resulting from surrounding mislocalized GP, Gram-negative bacteria regulate the GP contents from the outer leaflet through several mechanisms. PagP is an OM palmitoyltransferase that removes sn-1 palmitate residue (C16:0) from GP and transfers it to lipid A or PG, producing hepta-acylated Lipid A or palmitoylated PG with lysophospholipid as by-products (15, 16, 17). PagP is expressed at a low level in unstressed cells but can be induced by PhoPQ or membrane damage (18). Unlike PagP, another OM phospholipase PldA removes both sn-1 and sn-2 fatty acid side chains from GP and lysophospholipid that have miscompartmentalized to the outer leaflet of the OM (19). Moreover, it is known that accumulated GP in the OM can be translocated back to the IM by retrograde (OM to IM) GP transport to maintain the OM homeostasis. The Mla system consisting of an ABC transporter MlaFEDB, a periplasmic protein MlaC, and an OM lipoprotein MlaA has been shown to be a phospholipid transport system that is important for maintaining OM asymmetry (12, 20).

Studies on two-component systems including PhoPQ, PmrAB, and CrrAB, regulators of polymyxin resistance, mainly focused on LPS modification (21). However, our understanding of whether GP alterations vary with lipid A modification is still lacking. Mutations in *crrB* have only recently been reported to regulate high-level polymyxin resistance through CrrAB activation (22). Here we analyzed the GP alterations regulated by CrrAB using lipidomic analysis and found that CrrAB activated PagP, which appears to act as a bifunctional palmitoyltransferase that transfers palmitate chain from GP to both lipid A and PG within the OM, generating palmitoylated lipid A and acyl-PG in *K. pneumoniae*. The altered outer membrane may attenuate lipopolysaccharide activation of TLR4 signal transduction pathway and contribute to selective cationic antimicrobial peptide (CAMP) resistance. Our work presented that the phospholipid regulation varies with lipopolysaccharide modification, which provides insight into possible antimicrobial strategies that target the OM barrier.

## Materials and methods

### Bacterial strains and culture

Chromosomal deletions of *pagP*, *mlaC* were performed in the *crrB*^P151S^ mutant, which was a mutant of *K. pneumoniae* ATCC BAA 2146. *K. pneumoniae* cells were grown in LB broth at 37°C with shaking until the exponential phase (*A*_600_ 0.6–0.8). Minimum inhibitory concentrations (MICs) were determined in 96-well plates using a standard 2-fold microdilution method as described before (22).

### Gene editing with CRISPR-Cas9

Gene editing in *K. pneumoniae* was performed with CRISPR-Cas9 as previously described with some modifications (23). Wild-type sequences were analyzed via the CRISPOR website to identify appropriate N20 sequences (24). The N20 sequence was assembled into plasmid pSGKP-spe (Addgene, catalog#117234) by Golden gate assembly to form a gRNA cassette. The gRNA cassette and ssDNA template were electroporated into the competent cells with plasmid pCas-apr (Addgene, catalog#117231) for gene editing. All the primers used were shown in supplemental Table S1.

### Lipid A characterization with ESI-TOF-MS

Lipid A was isolated using mild acid hydrolysis as described before (22). The dried lipids were redissolved in chloroform/IPA/MeOH/H2O (1:1:1:0.5, v/v) and characterized by an U3000 HPLC system (Thermo Fisher) coupled to a Triple TOF 5600 mass spectrometer (AB Sciex) on negative ion mode with a mass range from 1,100 to 2,400 *m/z*. A volume of 2 μl of each sample was separated with ZORBAX RRHD 300 Å SB C8 (Agilent, 4.6x150 mm,5 μm) using a gradient consisting of mobile phase A (MeOH/acetonitrile/IPA, 1:1:1, 5 mM NH_4_OAC) and mobile phase B (MeOH/IPA, 1:4, 5 mM NH_4_OAC). The gradient started from 10% B to 60% B over 5 min, then to 100% B at 18 min, followed by a wash with 100% B for another 2 min, and re-equilibration for 5 min with 10% B at a flow rate of 1 ml/min. Data were acquired using an ion spray voltage of 4.5 kV, curtain gas of 35 PSI, nebulizer gas of 60 PSI, and an interface heater temperature of 600°C.

### The outer and inner membrane isolation

Outer and inner membrane fractions were isolated using a lysozyme/EDTA spheroplasting method and homogenization (25). Cells were resuspended in 8 ml of 0.5 M sucrose, 10 mM Tris pH 7.5. Lysozyme (20 μg/ml), phenylmethylsulfonyl (2 mM), and DNase I were added to the suspension, followed by the addition of 8 ml of 5 mM EDTA. Cells were homogenized by French Press (GLEN MILLS, America). Total membranes were isolated by centrifugation using an MLA-55 rotor in Optima MAX-XP Ultracentrifuge (Beckman) at 185,000 *g* for 1 h and resuspended in 20% (w/v) sucrose (1 mM EDTA, 1 mM Tris, pH 7.5). The inner and outer membranes were separated by the use of a defined 73%-53%–20% sucrose gradient through ultracentrifugation with an MLS-50 rotor (250,000 *g*, 16 h, 4°C). Western blots for OmpA (OM) and NADH dehydrogenase assay (IM) were used to assess the purity of the membranes.

### Lipidomic analysis

All experiments were conducted with four technical replicates for three biological replicates. Lipid extraction was performed with methyl-tert-butyl ether as described before with some modifications (26). Cell pellets were resuspended in 1×PBS and disrupted by ultrasonication (4 s/burst, ∼6 s between bursts, 2 min in total). An aliquot equal to 500 μg protein was used for lipid extraction after protein determination with a Pierce BCA protein assay kit (Thermo Fisher). Lipids were extracted with a double-phase solution (methyl-tert-butyl ether/MeOH/H2O, 10:3:2.5, v/v/v). Commercial 16:0-d31-18:1 PE, 16:0-d31-18:1 PC, 16:0-d31-18:1 PG, 16:0-d31-18:1 PS, C17 Ceramide (d18:1/17:0), 1,3-17:0 D5 diacylglycerol, D33-FFA 17:0 from Avanti lipids were added to the samples as internal standards for untargeted lipidomics, and 16:0-d31-18:1 PE, 16:0-d31-18:1 PG, 16:0 LPE-d9 were used as internal standards for targeted lipidomics. The upper phase was collected, dried under nitrogen, and redissolved in chloroform/MeOH (1:1, 10 mM NH4OAC) for LC/MS/MS. The pooled QC samples were injected during the batch analysis.

Untargeted lipidomic analysis was performed on Triple TOF 5600 (AB Sciex) equipped with a U3000 system (Thermo Fisher). Samples were separated with ACQUITY UPLC HSS T3 (Waters, 2.1×100 mm, 1.8 μm) using a multistep gradient that consisted of mobile phase A (MeOH/acetonitrile/H2O, 1:1:1, v/v, 5 mM NH4OAC) and mobile phase B (MeOH/IPA, 1:4, v/v, 5 mM NH4OAC). The gradient is from 10% B to 10% B within 1 min, 1 min–6 min to 60% B, 6 min–18 min to 100% B, then keep at 100% B for 2 min, followed by equilibration with 10% B for 5 min at a flow rate of 250 μl/min. Samples were analyzed by a full scan followed by 10 MS/MS scan from 50 to 1,250 *m/z* on positive and negative ion modes. The electrospray voltage was set as 5.5 kV in positive mode and 4.5 kV in negative ion mode. Nebulizer gas (GS1), heater gas (GS2), curtain gas (CUR) were separately set to 60, 60, 35 PSI, and interface heater temperature was set to 600°C. The collision energy was set to 35 ± 15 eV.

Targeted lipidomic analysis of PE, PG, acyl-PG, Lyso-PL was conducted on API 6500 Qtrap (AB Sciex) with multiple reaction monitoring (MRM) in negative ion mode. The LC condition was the same as described above. The Q1/Q3 transitions of the identified lipids were based on the MS2 spectra from Triple TOF 5600, which were described in supplemental Table S2.

### Data processing for lipidomics

Untargeted lipidomic analyses were performed through commercial software lipidview 1.3, peakview 2.1, and multiquant 3.0 (AB Sciex). Raw data of a pooled QC sample were input to lipidview 1.3 for lipid identification. Precursor and fragment mass tolerance was set to 10 ppm, and minimum signal-to-noise ratio of TOF peaks was set to 3. The identification result from lipidview 1.3 was checked with peakview 2.1 based on accurate mass, retention time, isotopic pattern, and MS2 spectrum. Samples was quantified based on the identification result with multiquant 3.0 using peak area and normalized to nM lipids/mg protein with internal standards. Lipids with a fold change above 1.5 and *P*-value below 0.05 were thought to be altered. Statistical analysis was performed with Student’s *t* test or one-way ANOVA with Tukey multiple comparisons on R software.

## Results

### CrrAB activation increases the level of acyl-glycerophosphoglycerols with long acyl chains

**Fig. 1:**
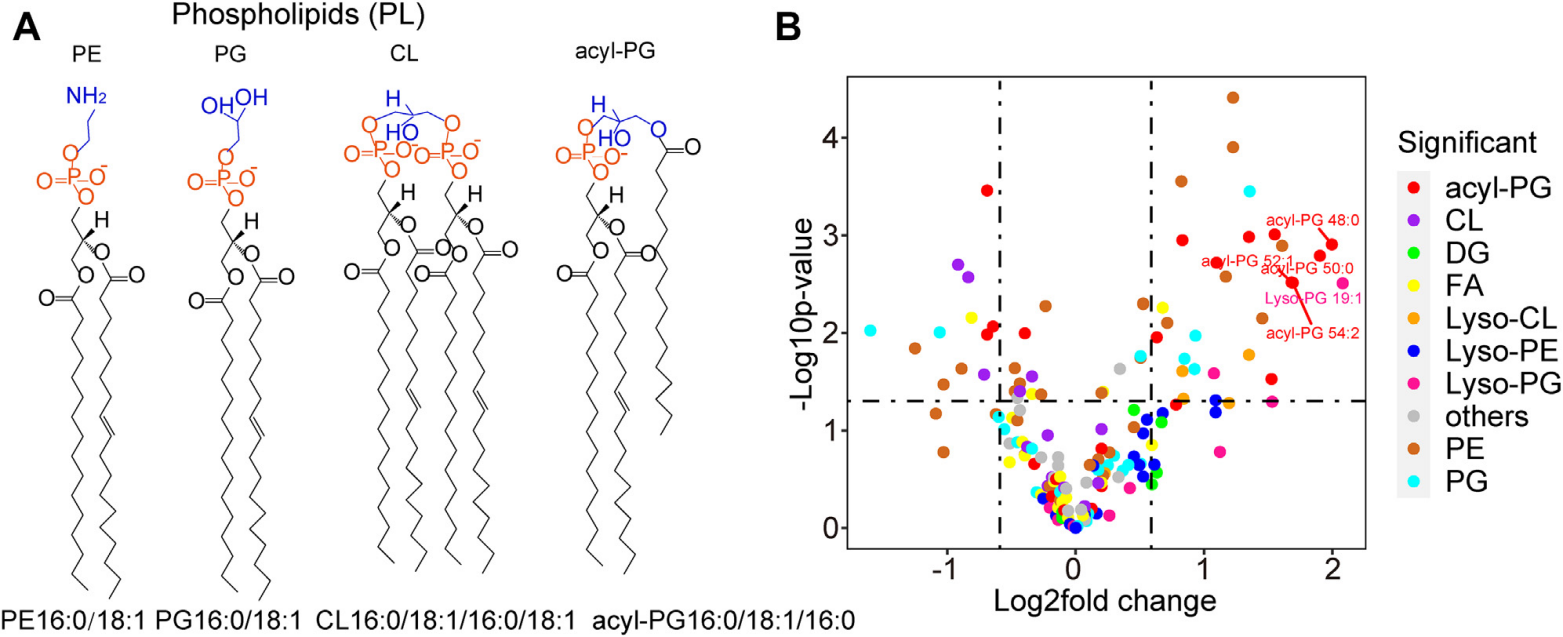
Lipidomic analysis of WT and *crrB* mutant based on ESI-TOF-MS. A: The representative structure of PE, PG, CL, and acyl-PG. B: The volcano plot indicated that the level of acyl-PG species, especially acyl-PG 48:0, acyl-PG 50:0, acyl-PG 54:2, and acyl-PG 52:1, were highly elevated in the *crrB* mutant in comparison with control. The experiment was repeated three times with four technical replicates each, and the data are given in supplemental File S2.

Mutations in the CrrAB two-component regulatory systems confer a high level of polymyxins resistance (27). *K. pneumoniae* ATCC BAA 2146 and its paired *crrB* mutant *crrB*^P151S^, which were reported previously, were employed to study the lipid alterations regulated by CrrAB activation (22). Untargeted lipidomic analysis of *K. pneumoniae* based on ESI-TOF-MS was built for lipid identification and quantification. The volcano plot showed that four of the top five altered lipids belonged to acyl-PG species, including acyl-PG 48:0 (3.99-fold), acyl-PG 50:0 (3.74-fold), acyl-PG 54:2 (3.23-fold), and acyl-PG 52:1 (3.21-fold) (Fig. 1B). Acyl-PG, modified PG molecules, are acylated with fatty acyl chains at the sn-3 position on the glycerol backbone of PG (Fig. 1A). Unlike predominant PE and PG in the membrane, the biosynthesis and function of acyl-PG have not been defined. In the study, we identified 22 kinds of acyl-PG in the sum composition level, which may contain different types of isomers differing in acyl chain compositions based on the fragmentation patterns in both positive and negative ion modes (Table 1 and supplemental Fig. S1). The alterations of acyl-PG showed that *crrB* mutant tended to synthesize acyl-PG with long acyl chains. For the saturated acyl-PG, the level of short-chain acyl-PG (C42-C44) was reducing or unvarying, while the level of longer chain acyl-PG (C46-C50) was increased at the sum composition level in the *crrB* mutant compared with wild type. Likewise, the relative abundance of acyl-PG with one, two, or three of double bonds in the attached fatty acids presented a similar tendency as the increasing number of carbons (Table 1).

**Table 1:** The alterations of acyl-PG species annotated based on ESI-TOF-MS

Sum Composition	Molecular lipid Information	FC^a^	*P*-value
acyl-PG 42:0	acyl-PG 14:0_16:0/12:0	0.80	2.20E-01
acyl-PG 44:0	acyl-PG 14:0_16:0/14:0 + acyl-PG 16:0_16:0/12:0 + acyl-PG 12:0_16:0/16:0	1.09	6.33E-01
acyl-PG 46:0	acyl-PG 14:0_16:0/16:0	2.14 ↑	1.91E-03
acyl-PG 48:0	acyl-PG 16:0_16:0/16:0	3.99 ↑	1.24E-03
acyl-PG 50:0	acyl-PG 16:0_18:0/16:0	3.74 ↑	1.62E-03
acyl-PG 42:1	acyl-PG 14:0_16:1/12:0	1.15	1.54E-01
acyl-PG 44:1	acyl-PG 16:0_16:1/12:0	0.62 ↓	3.47E-04
acyl-PG 46:1	acyl-PG 16:0_16:1/14:0 + acyl-PG 16:0_18:1/12:0 + acyl 14:0_16:1/16:0 + acyl 14:0_16:0/16:1	0.90	3.16E-01
acyl-PG 48:1	acyl-PG 16:0_16:1/16:0	1.78 ↑	1.12E-03
acyl-PG 50:1	acyl-PG 16:0_18:1/16:0	2.93 ↑	9.80E-04
acyl-PG 52:1	acyl-PG 18:0_18:1/16:0	3.21 ↑	3.03E-03
acyl-PG 44:2	acyl-PG 14:0_14:1/16:1 + acyl-PG 12:0_16:1/16:1 + acyl-PG 12:0_14:1/18:1	0.76	1.01E-02
acyl-PG 46:2	acyl-PG 16:1_18:1/12:0 + acyl-PG 14:0_18:1/14:1 + acyl-PG 14:0_16:1/16:1	0.64 ↓	8.60E-03
acyl-PG 48:2	acyl-PG 16:0_16:1/16:1	0.94	6.60E-01
acyl-PG 50:2	acyl-PG 16:0_18:1/16:1 + acyl-PG 16:1_18:1/16:0 + acyl-PG 16:0_16:1/18:1	1.55 ↑	1.11E-02
acyl-PG 52:2	acyl-PG 16:0_18:1/18:1 + acyl-PG 18:1_18:1/16:0	2.55 ↑	1.04E-03
acyl-PG 54:2	acyl-PG 18:1_18:0/18:1	3.23 ↑	3.06E-03
acyl-PG 46:3	acyl-PG 16:1_16:1/14:1	0.62 ↓	1.04E-02
acyl-PG 48:3	acyl-PG 16:1_18:1/14:1 + acyl-PG 16:1_16:1/16:1	0.88	4.74E-01
acyl-PG 50:3	acyl-PG 16:1_18:1/16:1	1.15	3.73E-01
acyl-PG 52:3	acyl-PG 16:1_18:1/18:1 + acyl-PG 18:1_18:1/16:1	1.72	5.44E-02
acyl-PG 54:3	acyl-PG 18:1_18:1/18:1	2.88 ↑	2.97E-02

a The fold change of the lipid levels in the *crrB* mutant compared with the wild type. Data are given in supplemental File S2.

### CrrAB activates the expression of palmitoyltransferase PagP and Mla system proteins

**Fig. 2:**
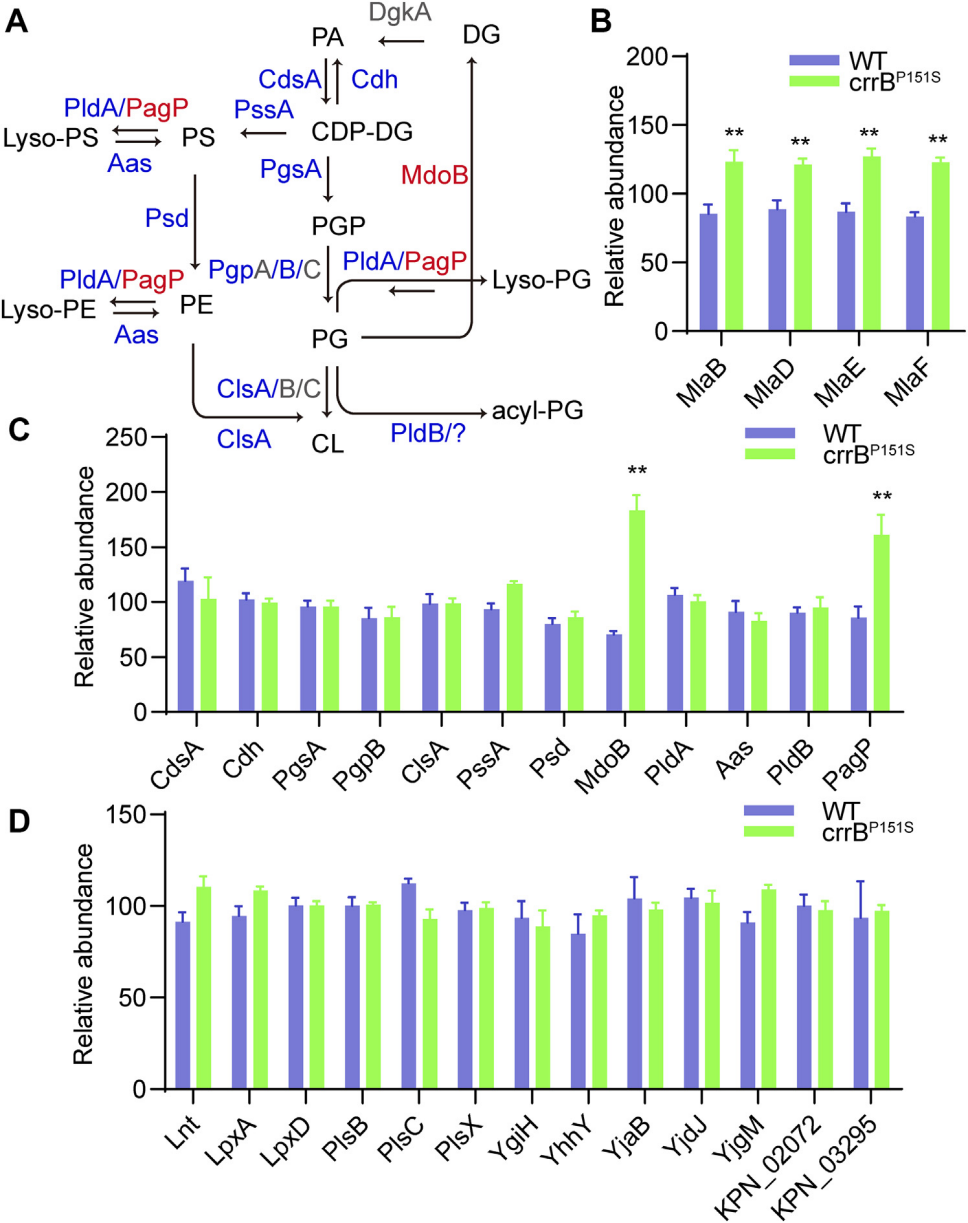
CrrAB activation led to an increased level of palmitoyltransferase PagP and Mla system proteins. A: The identified biosynthetic and metabolic pathway of bacterial phospholipid. Proteins in blue, red, and gray, respectively, represented proteins that were unaltered, upregulated, and unidentified in the *crrB* mutant compared with the wild type. B: The expression of Mla system proteins including MlaB, MlaD, MlaE, and MlaF were upregulated in the *crrB* mutant. C: The expression of enzymes that mediate the synthesis and metabolism of phospholipids in *K. pneumoniae*. D: The expression of acyltransferases identified in the *K. pneumoniae* that were hypothesized to mediate the synthesis of acyl-PG had no difference between WT and *crrB* mutant. The experiment was repeated three times with two technical replicates each and the proteomic data are available via ProteomeXchange with identifier PXD018528.

CrrAB activation leads to an increase of long-chain acyl-PG, which must be associated with the synthesis and metabolism of GP. In order to uncover the determinant, we screened the identified enzymes that mediate the synthesis and metabolism of GP in our previous proteomic data (Fig. 2A, C) (22). Considering that the synthesis of acyl-PG is not fully understood, we also sorted out the acyltransferases identified in *K. pneumoniae* including Lnt, LpxA, LpxD, PlsB, PlsC, PlsX, YgiH, YhhY, YjaB, YjdJ, YjdM, KPN_02072, and KPN_03295 that were hypothesized to transfer an acyl chain from GP to PG, forming acyl-PG (Fig. 2D). Of all enzymes mentioned above, only palmitoyltransferase PagP (1.92-fold) and phosphoglycerol transferase I MdoB (2.60-fold) were upregulated in the *crrB* mutant compared with the wild type (Fig. 2C). MdoB has no correlation with the production of acyl-PG. Unlike MdoB, PagP has been reported as a lipid A palmitoyltransferase, and we hypothesized that the PagP may transfer an acyl chain to PG to produce acyl-PG, leading to accumulated acyl-PG. Besides, the expression of Mla proteins including MlaB (1.40-fold), MlaD (1.35-fold), MlaE (1.47-fold), MlaF (1.47-fold), which were thought to be involved in lipid trafficking, were upregulated in the *crrB* mutant (Fig. 2B) (28). As GP is synthesized on IM, and then transported to OM, upregulated Mla system complex may play a role in acyl-PG generation as GP transporter.

### Elevated long-chain acyl-PG contents attributed to the upregulated palmitoyltransferase PagP in the *crrB* mutant

**Fig. 3:**
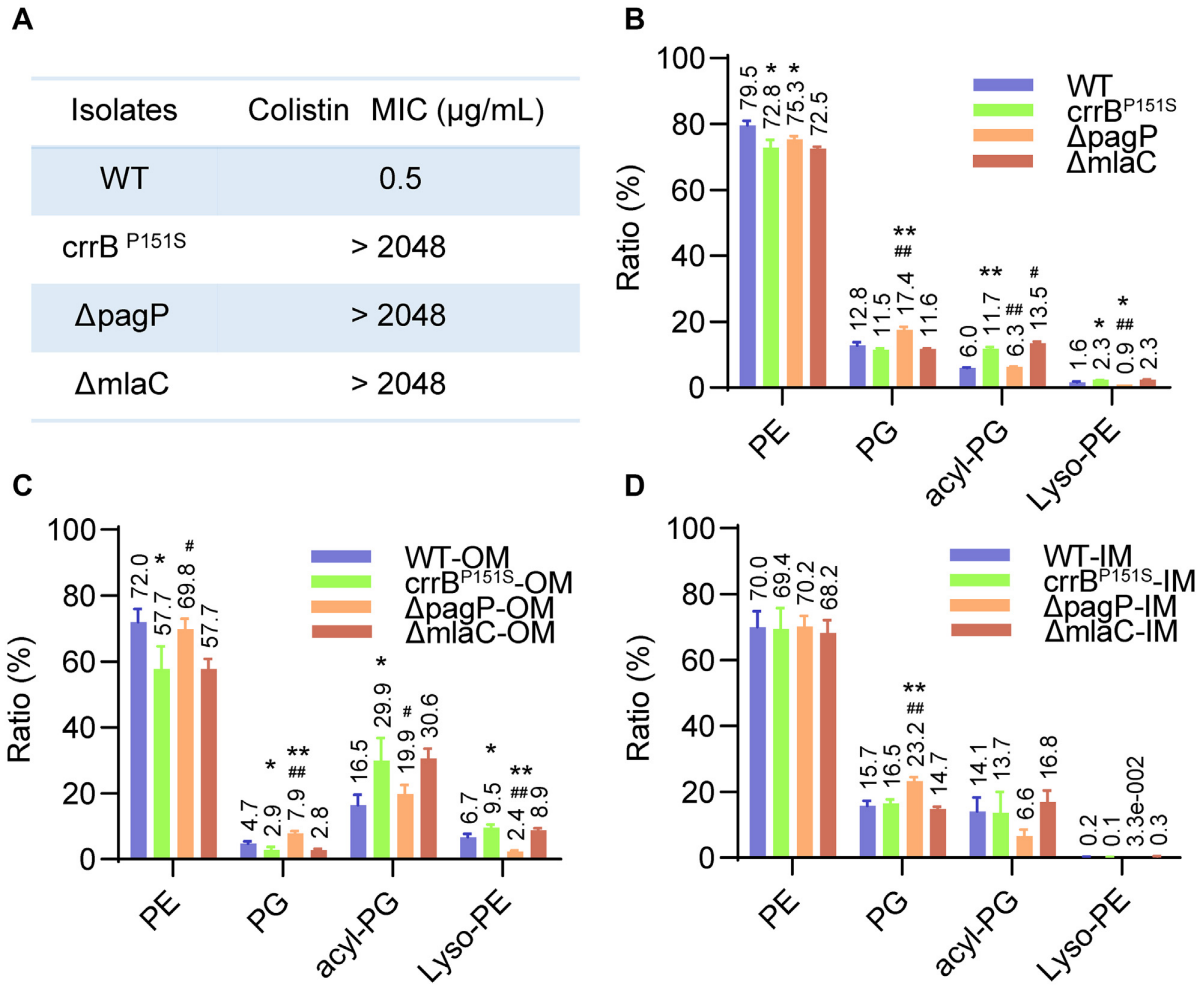
The alterations of the relative abundance among each GP species. A: The colistin MIC of strains. B: Each GP percentage of the total lipid amount detected by ESI-MRM-MS. C: Each GP percentage of total lipid amount within the OM. D: Each GP percentage of total lipid amount within the IM. ∗(*P*-value<0.05) or ∗∗(*P*-value<0.01) indicates a significance of *crrB*^*P151S*^, *ΔpagP*, or *ΔmlaC* compared with WT. #(*P*-value<0.05) or ##(*P*-value<0.01) shows a significance of *ΔpagP* or *ΔmlaC* in comparison with *crrB*^*P151S*^. The *pagP* and *mlaC* deletions were constructed in the *crrB*^P151S^ mutant.

To explore it, we knocked out the *pagP* and *mlaC* gene of the Mla system in the *crrB* mutant by CRISPR-Cas9, respectively. PagP and MlaC were not the determinants of polymyxin resistance since the colistin MIC showed no difference before and after the gene knockout in the *crrB* mutant (Fig. 3A). Targeted lipidomic analysis of acyl-PG, PE, and PG from strains including WT, *crrB* mutant, Δ*pagP*, and Δ*mlaC* based on MRM was conducted according to the results of untargeted lipidomics. The results showed that the acyl-PG percentage of the total amount was significantly reduced after *pagP* knockout. Simultaneously, the ratio of PG species was elevated and that of lyso-PE was sharply diminished. Unlike *PagP*, the removal of *mlaC* brought minor changes to GP species (Fig. 3B).

**Fig. 4:**
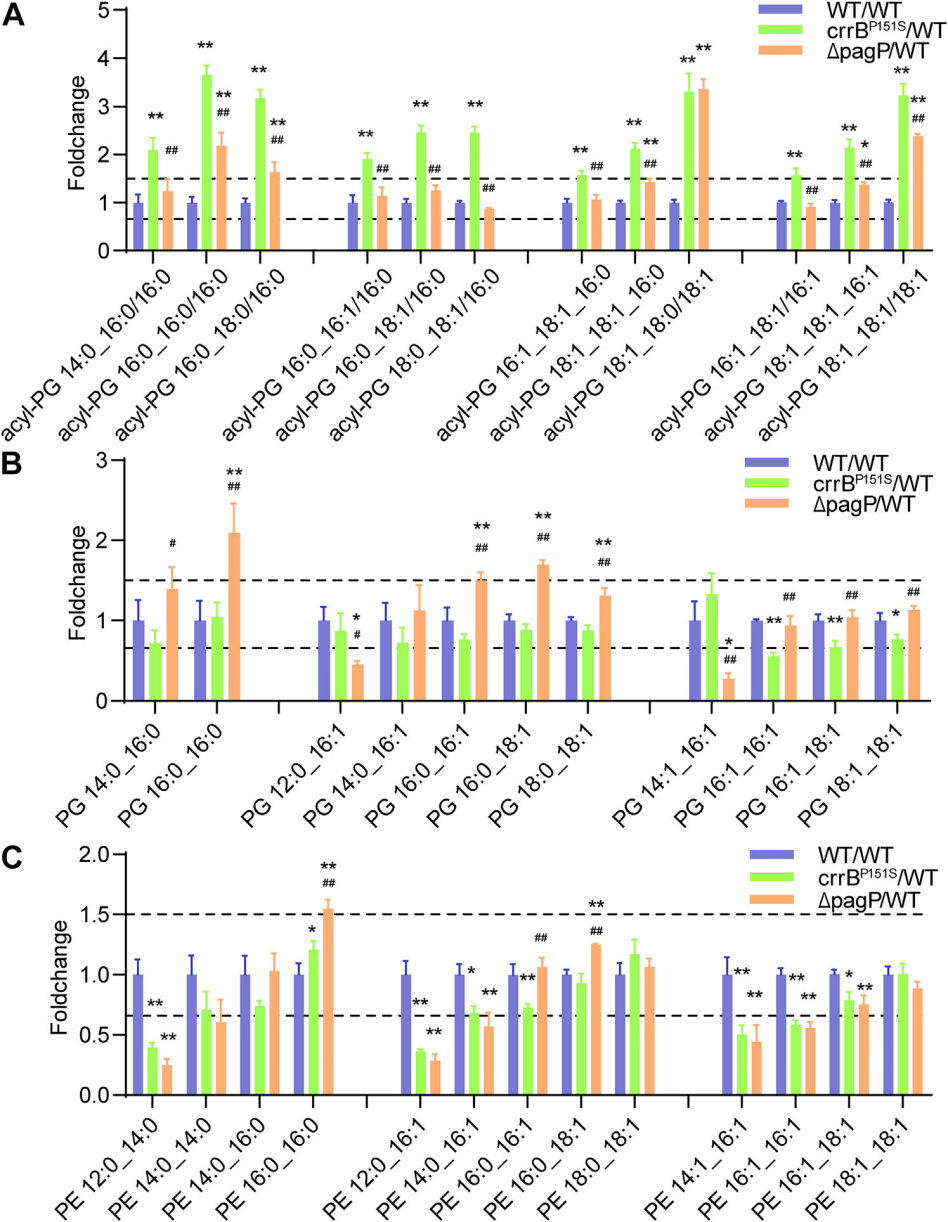
The alterations of acyl-PG, PG, and PE of whole-cell lysates. A: Of the 12 increased acyl-PG in the *crrB* mutant, 11 were decreased after *pagP* knockout. The level of acyl-PG 18:1_18:0/18:1 and acyl-PG 18:1_18:0/18:1 showed a relatively minor difference before and after *pagP* knockout. B: PG alterations in the *crrB* mutant before and after *pagP* knockout. C: The alterations of PE with and without a palmitate chain in the *crrB* mutant before and after *pagP* knockout. Dashed lines marked the fold change threshold of 1.5. The experiment was repeated three times with four technical replicates each, and the data are given in supplemental File S3. ∗(*P*-value<0.05) or ∗∗(*P*-value<0.01) indicates a significance of *crrB*^*P151S*^ or *ΔpagP* compared with WT. #(*P*-value<0.05) or ##(*P*-value<0.01) shows a significance of *ΔpagP* in comparison with *crrAB*^*P151S*^.

PagP is a palmitoyltransferase, which was reported to transfer a palmitate chain to lipid A (29). Interestingly, most of the elevated acyl-PG with longer acyl chains in the *crrB* mutant owned C16:0 acyl moiety at sn-3. Acyl-PG 16:1_18:1_16:0, acyl 18:1_18:1_16:0, and acyl-PG 18:1_18:1_16:1 had several isomers differing in acyl chain of sn-3 position (Fig. 4A and Table 1). After *pagP* knockout, all of the acyl-PG with C16:0 or C16:1 at the sn-3 position appeared to be diminished, while acyl-PG (18:1_18:1)_18:0 (1.02-fold) and acyl-PG (18:1_18:1)_18:1 (0.74-fold) showed a minor difference (Fig. 4A). At the same time, the removal of *pagP* brought an increase in most of the PG, which may regard as acyl chain acceptors to producing acyl-PG. However, the level of PG 12:0_16:1 and PG 14:1_16:1 were reduced after *pagP* knockout, which was in accordance with the results that no acyl-PG using PG 12:0_16:1 and PG 14:1_16:1 as acyl chain acceptor were identified (Fig. 4B and Table 1). Furthermore, the *crrB* mutant showed a reduction in the level of PE with short acyl chains like PE 12:0_14:0, PE 12:0_16:1, PE 14:0_16:1, PE 16:0_16:1, PE 14:1_16:1, PE 16:1_16:1, PE 16:1_18:1. PagP inactivation in the *crrB* mutant had a minor impact on nonpalmitoylated PE contents, but lead to an increase of palmitoylated PE like PE 16:0_16:0, PE 16:0_16:1, and PE 16:0_18:1 (Fig. 4C). Thus, PagP may transfer palmitate from PE to PG, resulting in accumulated acyl-PG in the *crrB* mutant.

### CrrAB remodels the lipid components within the OM through PagP

As we know, PagP is an outer membrane protein. To further explore the extent of variations in the IM and OM content, lipidomic analyses of IM and OM contents based on MRM were conducted. The purity of the IM and OM were assessed by NADH dehydrogenase assay (IM) and Western blot of OmpA (OM), suggesting that the IM and OM were separated successfully (supplemental Fig. S2). The inner and outer membranes exhibit different GP distributions. As we can see, the level of lyso-PE and the ratio of acyl-PG to PG on the OM was much higher than that on the IM (Fig. 3C, D). The variations of GP caused by CrrAB activation mainly focused on the OM, which could be recovered after *pagP* knockout (Fig. 3C, D). However, the removal of *mlaC* brought a minor difference on the IM and OM in the *crrB* mutant (Fig. 3C, D).

**Fig. 5:**
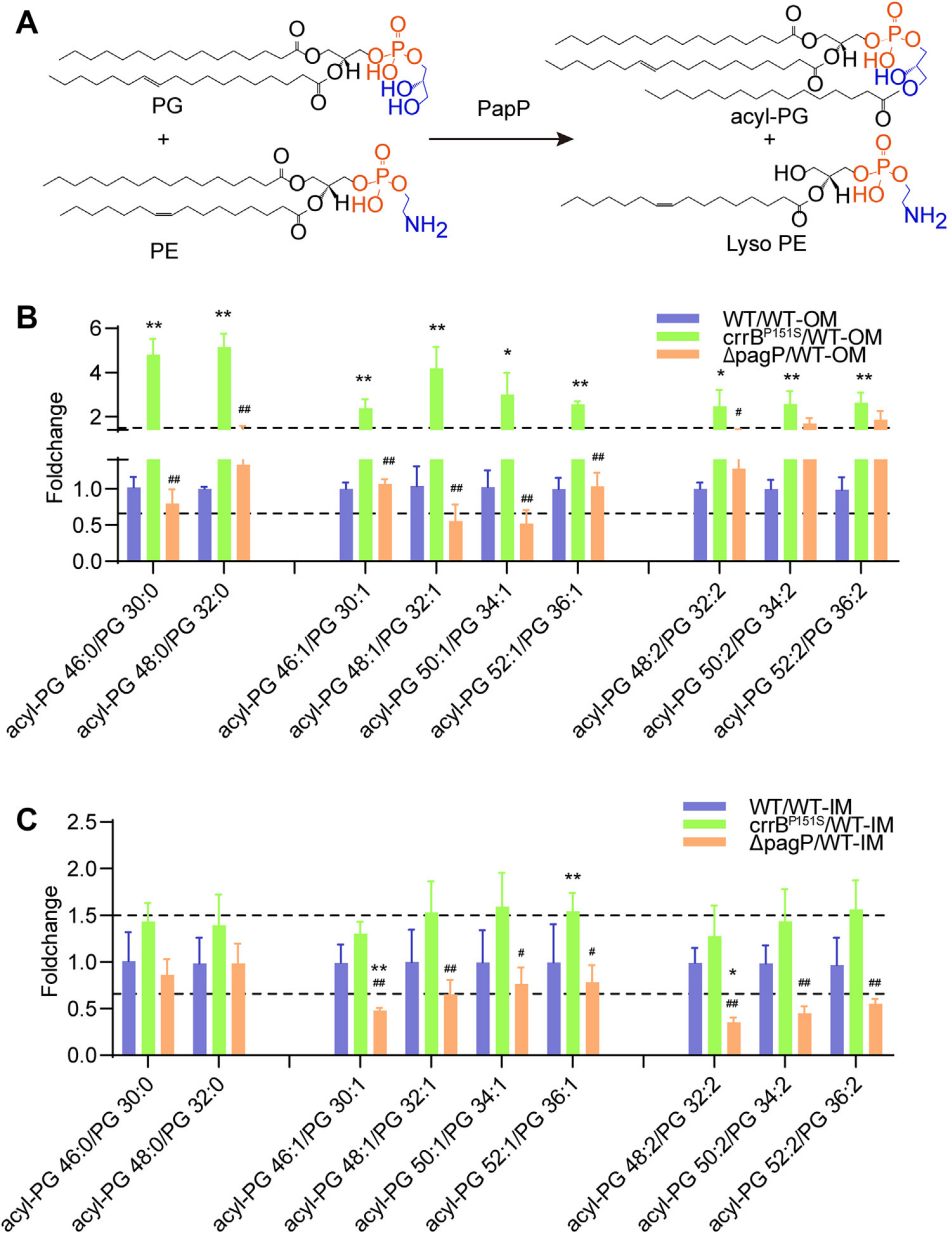
The alterations of lipid contents within the outer and inner membranes. A: The hypothesis of the generation of acyl-PG mediated by PagP mediator. B: The ratio of OM acyl-PG to its accordingly PG was significantly increased in the *crrB* mutant compared with the wild type, and decreased after *pagP* knockout within the outer membrane. C: The ratio of IM acyl-PG to its accordingly PG substrate showed a minor variance of *crrB* mutant compared with the WT, but significantly decreased in the *pagP* knockout group. The experiment was repeated three times with four technical replicates each, and the data are given in supplemental File S4. ∗(*P*-value<0.05) or ∗∗(*P*-value<0.01) indicates a significance of *crrB*^*P151S*^ or *ΔpagP* compared with WT. #(*P*-value<0.05) or ##(*P*-value<0.01) shows a significance of *ΔpagP* in comparison with *crrAB*^*P151S*^.

As the results mentioned above, we hypothesized that PGs are the acceptor substrates of PagP, which produce acyl-PG after receiving a palmitate chain (C16:0) (Fig. 5A). To reduce the deviation from the amount of membrane protein caused by CrrAB activation and PagP removal, the ratio of products to substrates like acyl-PG 48:1 (acyl-PG 16:0_16:1/16:0) to PG 32:1 (PG 16:0_16:1) was employed to assess the effect of PagP on the IM and OM. If the expression of PagP was upregulated, the ratio of products to substrates would be increased; otherwise, it would be decreased. CrrAB activation had a minor impact on the ratio of acyl-PG to PG, but PagP defect significantly reduced the ratio of acyl-PG to PG within the IM (Fig. 5C). Distinct from the IM, the ratio of OM acyl-PG to its accordingly PG substrate in the *crrB* mutant, like predominant acyl-PG 48:1 to PG 32:1 and acyl-PG 50:1 to PG 34:1, was sharply increased compared with the wild type. When the *pagP* gene was knocked out, most of them were reduced toward the level of the wild type (Fig. 5B). The alterations of OM PE were similar to that of the whole cells, while that of IM had minor variations (supplemental Fig. S3). Therefore, OM palmitoylated PE may be the donor substrates of the palmitate chains. Our results showed that CrrAB activation mainly remodels the GP contents within the OM other than IM, which depends on the PagP functioning on the OM.

### PagP is responsible for the lipid A palmitoylation

**Fig. 6:**
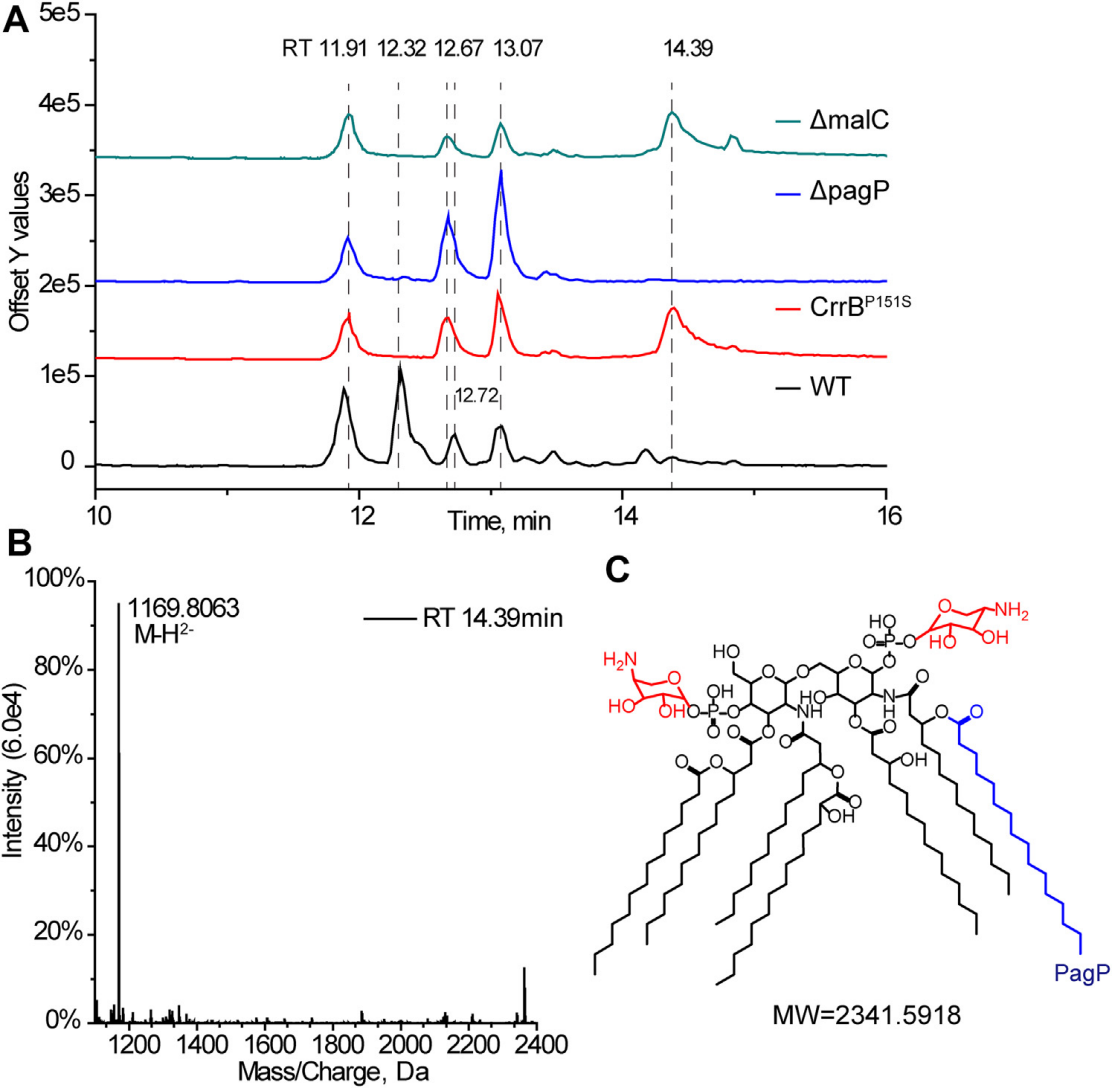
Lipid A analysis based on LC/MS. A: The base peak of lipid A in each strain. B: The corresponding mass spectrum at the retention time of 14.39 min. The ion at *m/z* 1169.8063 was doubly deprotonated. C: The corresponding lipid A structure at the retention time of 14.39 min represented lipid A with two L-Ara4N and a palmitoylation.

CrrAB activation induces lipid A modification with L-Ara4N and palmitoylation (22). In the study, extracted lipid A in the wild type was separated into four peaks on reversed-phase HPLC with retention times of 11.91, 12.32, 12.72, and 13.07 min, while that of the *crrB* mutant was separated into four peaks with retention times of 11.91, 12.67, 13.07, and 14.39 min (Fig. 6A). Specific fractions of the wild type at 12.32 and 12.72 min, which presented ions of *m/z* 1760.1693 and 1744.1822 in mass spectrum, respectively, correspond to the lipid A without modification (supplemental Fig. S4). Specific fractions of the *crrB* mutant at 12.67 and 14.39 min, whose corresponding ions were at *m/z* 2102.3389 and 1169.8063, respectively, represented lipid A with two L-Ara4N molecules and lipid A modified with two L-Ara4N and a palmitoylation (Figs. 6 and S4). Compared with the base peak of *crrB* mutant with *pagP*, the *pagP* knockout strain lost the peak at 14.39 min, which revealed that lipid A dropped the palmitate chain, corresponding to the previous study that PagP transfers a palmitate chain from GP to lipid A (29). Moreover, the lipid A profile of the *crrB* mutant showed little difference before and after the *mlaC* gene knockout (Fig. 6A). Therefore, the palmitoylation of lipid A in the *crrB* mutant was indeed mediated by *pagP*, which did not rely on the Mla system.

## Discussion

Recent advances have revealed that CrrAB activates LPS modification including L-Ara4N and phosphoethanolamine, leading to polymyxin resistance (22, 27, 30). Here we investigated that CrrAB activation not only stimulates LPS remodeling but also regulates the GP components especially acyl-PG species to maintain a balance within the OM.

It is of note that, although LPS was modified by L-Ara4N and palmitoylation via elevated expression of *arnBCADTEF* and *pagP*, CrrAB activation appeared to have a minor impact on the LPS biosynthesis and degradation pathway since the expression of proteins linked to LPS biosynthesis (LpxACDHBK/WaaA/LpxL), transport (LptACDE), and degradation (YciM/FtsH) remained stable compared with the wild type (data not shown) (31, 32). As a consequence, CrrAB activation regulated the ratio of modified LPS to unmodified LPS but presumably did not alter the total LPS level. Since lipid A modified with two L-Ara4N and a palmitoylation was the last-eluted on reversed-phase HPLC, the production of palmitoylated lipid A increased the hydrophobicity of the outer leaflet in the OM. Meanwhile, the ratio of acyl-PG species to PE and PG species in the *crrB* mutant was significantly elevated with the OM (∼2-fold), which increased the hydrophobicity of the inner leaflet in the OM. Interestingly, GP contents within the IM were almost invariable. Thus, the increased acyl-PG within the OM was thought to cooperate with the palmitoylated lipid A to maintain the balance of OM. The alteration of GP in the inner leaflet and the modified LPS in the outer leaflet increases the hydrophobicity of the OM. Previous studies have reported that elevated hydrophobicity could block surface association and membrane intercalation of amphipathic alpha-helical CAMP, promoting resistance to various CAMPs such as C18G (7, 33). According to this, elevated acyl-PG accompanied with palmitoylated lipid A in the *crrB* mutant may cocontribute to certain CAMP resistance. It is of note that the palmitoylation of lipid A caused by PagP was not the determinant of polymyxin resistance since the polymyxin MIC was still above 2048 μg/ml after blocking the palmitoylation of lipid A and PG by *pagP* knockout in the *crrB* mutant. PagP is required for *Bordetella bronchiseptica* to persist in the mouse respiratory tract (34) and to resist antibody-mediated complement lysis (35). Previous studies reported that PagP-mediated lipid A palmitoylation can attenuate LPS activation of the TLR4/MD2/CD14 innate immune receptor, conferring resistance to host immune defenses (36, 37, 38), which was in accordance with that *crrB* mutants display an increased in vivo virulence (27).

Acyl-PG, also known as hemi bis(monoacylglycero) phosphate, is a specific lipid species with undefined biosynthesis and function (39). Our results showed that acyl-PG species are enriched in membrane especially OM in *K. pneumoniae* (Fig. 3C, D). Combined with the untargeted and targeted lipidomics, we presented the acyl-PG profiles of *K. pneumoniae*, including 22 kinds in bond levels with more fatty acyl compositions by fragmentation patterns in both positive and negative ion modes (Table 1). The fragmentation patterns of acyl-PG in negative ion mode were already described by Hsu FF *et al.* and Coulon D *et al.*, which offered the fragment ion of the fatty acid chains at sn-1, sn-2, or sn-3 (40, 41). However, the corresponding position of fatty acid chains was difficult to determine. Here we found that the molecular ion [M+Na^+^] of acyl-PG fragmented at the linkage between phosphate and a carbon atom on both sides, producing ions [R_3_COOCH2CH(OH)CH2OPO3HNa]^+^ and [R_1_COOR_2_COOCH2CH(OH)CH2OPO3HNa]^+^, offering the information of fatty acid chain at sn-3 position (supplemental Fig. S1). The increased level of acyl-PG in the *crrB* mutant backing toward the wild type after *pagP* knockout revealed that PagP is one of the enzymes responsible for acyl-PG generation, which corresponds to a previous study that purified PagP transfers palmitate to PG (16). PagP is a lipid A palmitoyltransferase possessing precise catalyzation activated by PhoPQ or OM damage (17). The different alterations of palmitoylated and nonpalmitoylated PE before and after the removal of PE are corresponding to the results that PagP can distinguish palmitate from other acyl chains found in GP (29) (Fig. 4C). Considering that PagP was an OM protein remodeling the distribution of OM lipids, acyl-PG was thought to be synthesized in the OM (42). The decreased acyl-PG ratio and increased PG ratio within the IM after *pagP* knockout suggested that enriched OM acyl-PG may translocate to IM and PG synthesized in the IM may transfer to OM as substrates when PagP was activated (Fig. 3D). To date, the pathways for anterograde and retrograde transport of GP are less understood. The Mla system has been implicated in both retrograde and anterograde GP transport (43, 44). Mla proteins including MlaB, MlaD, MlaE, MlaF were overexpressed in the *crrB* mutant (Fig. 2B). However, the GP contents including PE, PG, acyl-PG, Lyso-PE within the OM and IM in the *crrB* mutant lack significant changes in the defect of the Mla system. Thus, we suspected that the Mla pathway targets a relatively minor population of OM GP molecules or there were unknown lipid trafficking systems offsetting the function of the Mla system in the process of CrrAB activation. Previous studies have reported that the MsbA-dependent flip of lipids from the IM to the OM is required for lipid A palmitoylation in vivo (18) and it has been demonstrated to possess phospholipid flippase activity in vitro using fluorescent phospholipid derivatives (45). Since a previous study reported that the active site of PagP was located on the outer leaflet of the OM, phospholipids like PG and PE, normally found in the inner leaflet, may translocate to the outer leaflet as substrates, and acyl-PG products were transported to the inner leaflet when CrrAB was activated (42).

Except for acyl-PG, phospholipids including PE, PG, lyso-PE altered in the *crrB* mutant to some extent. Interestingly, the levels of acyl-PG (18:1_18:1)_18:0 and acyl-PG (18:1_18:1)_18:1 that should not be affected by the expression of PagP were elevated in the *crrB* mutant. Furthermore, the level of PE without palmitate like PE 12:0_14:0, PE 12:0_16:1, PE 14:0_16:1, PE 14:1_16:1, PE 16:1_16:1, PE 16:1_18:1 that should not affected by PagP was diminished in the *crrB* mutant. However, the expression of proteins belonging to de novo pathway of PE, PG, and CL like CdsA, PssA, Psd, PgsA, PgpA/B/C, ClsA, PldA showed no difference between the *crrB* mutant and the wild type. Hence, in order to keep a balance between membrane integrity and lipid A modification, CrrAB activation may modulate the GP contents through other pathways.

Our study demonstrates that the CrrAB two-component system regulated lipid A palmitoylation and the outer membrane GP through elevated palmitoyltransferase PagP in *K. pneumoniae*. Although studies about PagP are extensive, most studies focused on lipid A palmitoylation. Our study suggests that PagP acts as a bifunctional palmitoyltransferase to PG and lipid A, contributing to acyl-PG generation. The finding broadens our understanding of PL metabolism and regulation, assisting in new therapeutic strategies targeting on OM barrier.

## Data availability

Proteomic data are available via ProteomeXchange with identifier PXD018528.

## Supplemental data

This article contains supplemental data

## Conflicts of interest

The authors declare that they have no known competing financial interests or personal relationships that could have appeared to influence the work reported in this paper.

## Supplemental data

Supplementary file 1 revised
