# Supplementary material for: CrrAB regulates PagP-mediated glycerophosphoglycerol palmitoylation in the outer membrane of Klebsiella pneumoniae
Source: J Lipid Res. 2022 Jul 13;63(9):100251. doi: 10.1016/j.jlr.2022.100251 (PMC9403492; doi:10.1016/j.jlr.2022.100251)
Supplement: Supplementary file 1 revised [file mmc1.pdf]

# CrrAB regulates PagP-mediated glycerophosphoglycerol palmitoylation in the outer membrane of *Klebsiella pneumoniae*

**Lang Sun [1], Youwen Zhang [1], Tanxi Cai [2,3], Xue Li [1], Na Li [2,3], Zhensheng Xie [2,3], Fuquan Yang [2,3*], Xuefu You [1*]**

[1]Beijing Key Laboratory of Antimicrobial Agents, Institute of Medicinal Biotechnology, Chinese Academy of Medical Sciences & Peking Union Medical College, Beijing 100050, China.

[2]Key Laboratory of Protein and Peptide Pharmaceuticals & Laboratory of Proteomics, Institute of Biophysics, Chinese Academy of Sciences, Beijing 100101, China.

[3]University of Chinese Academy of Sciences, Beijing 100049, China.

***Corresponding author:**

Fuquan Yang, Email: fqyang@ibp.ac.cn;

Xuefu You, Email: xuefuyou@imb.pumc.edu.cn.

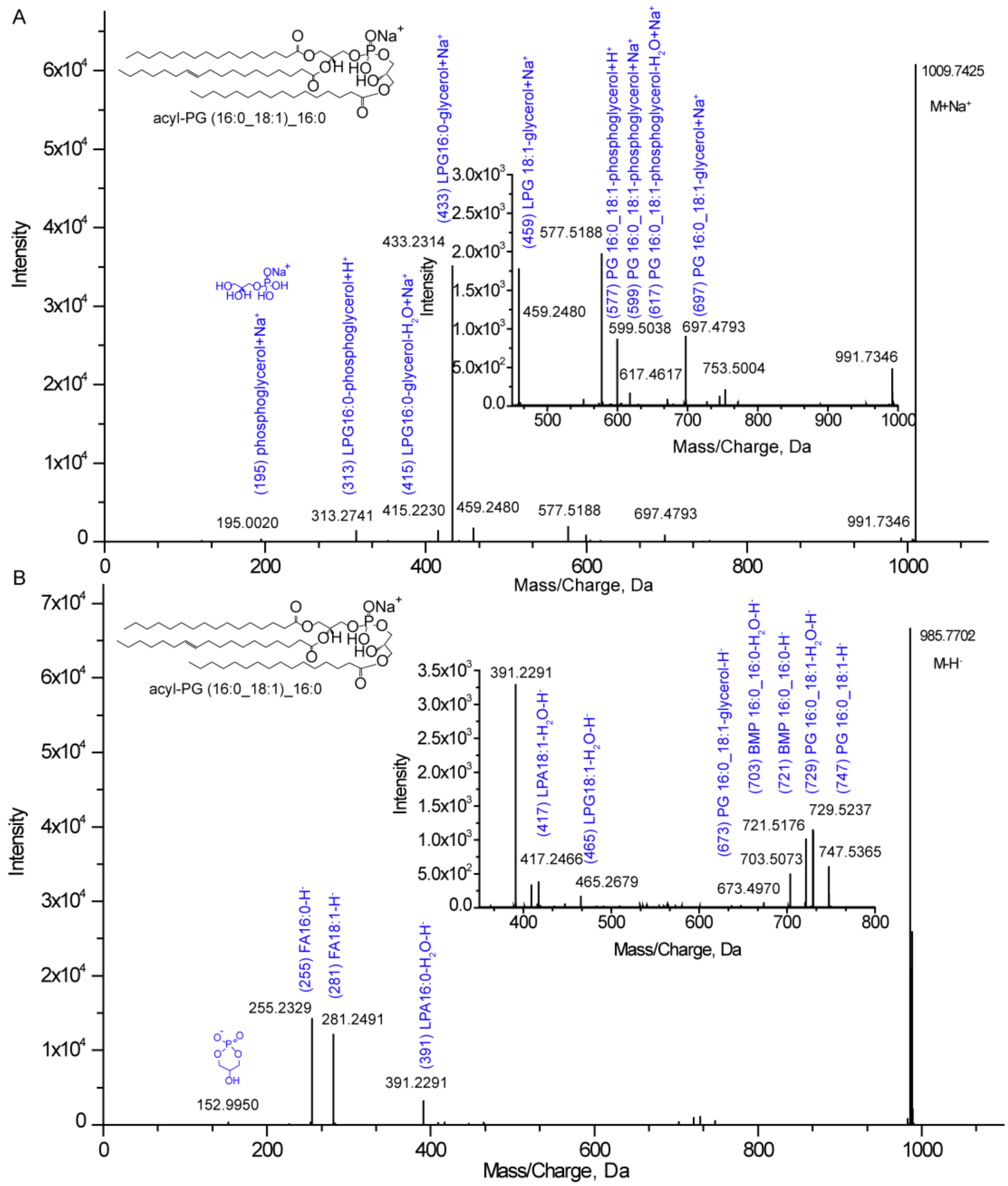


Fig. S1. The representative annotation of acyl-PG based on MS2 spectra in both positive (A) and negative (B) modes.

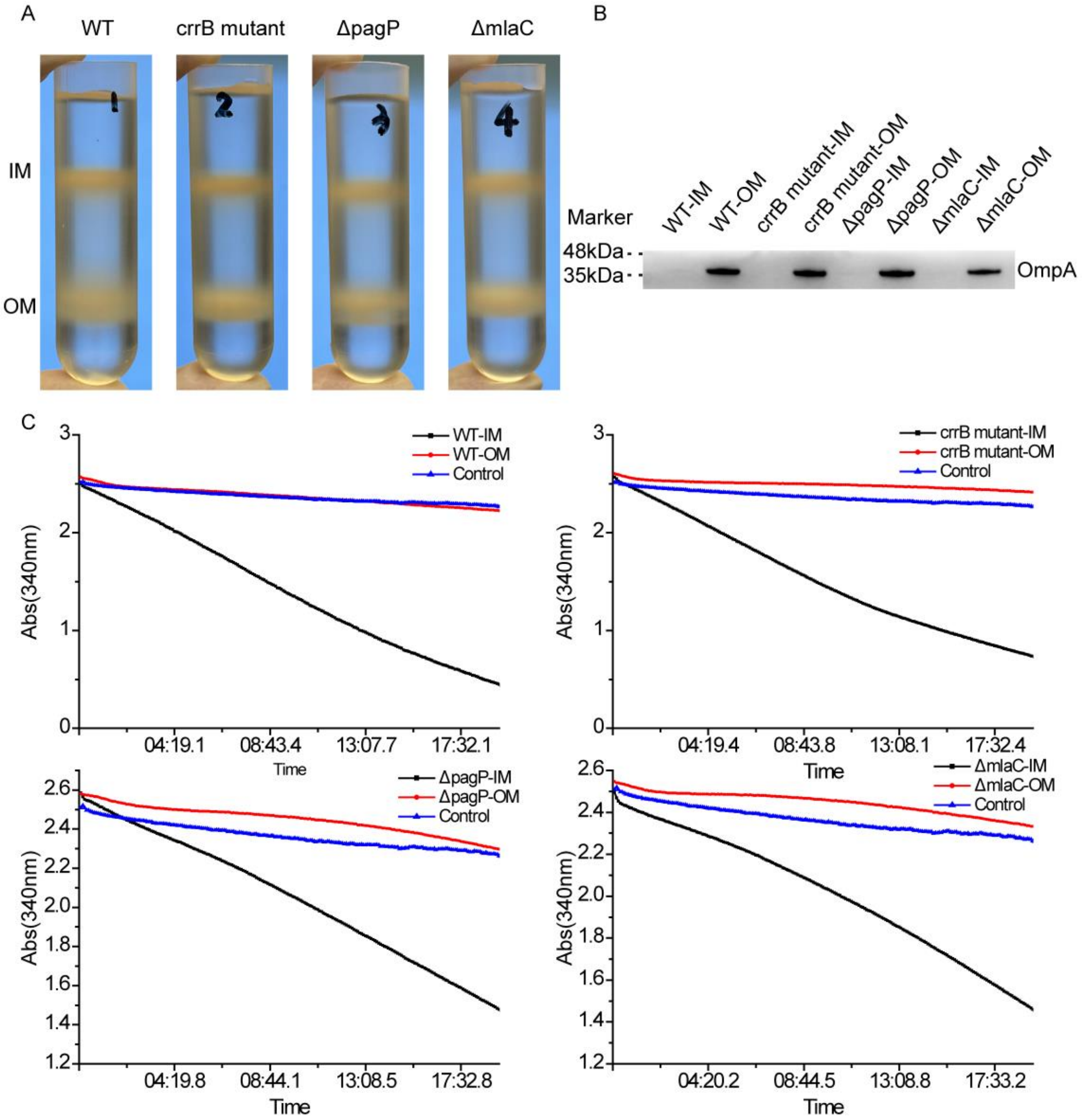


Fig. S2. The purity of IM and OM separation. (A) Representative results for the separation of IM and OM. (B) Western blot for the OM-localized OmpA protein indicated the purity of IM. (C) NADH dehydrogenase assay revealed the purity of OM.

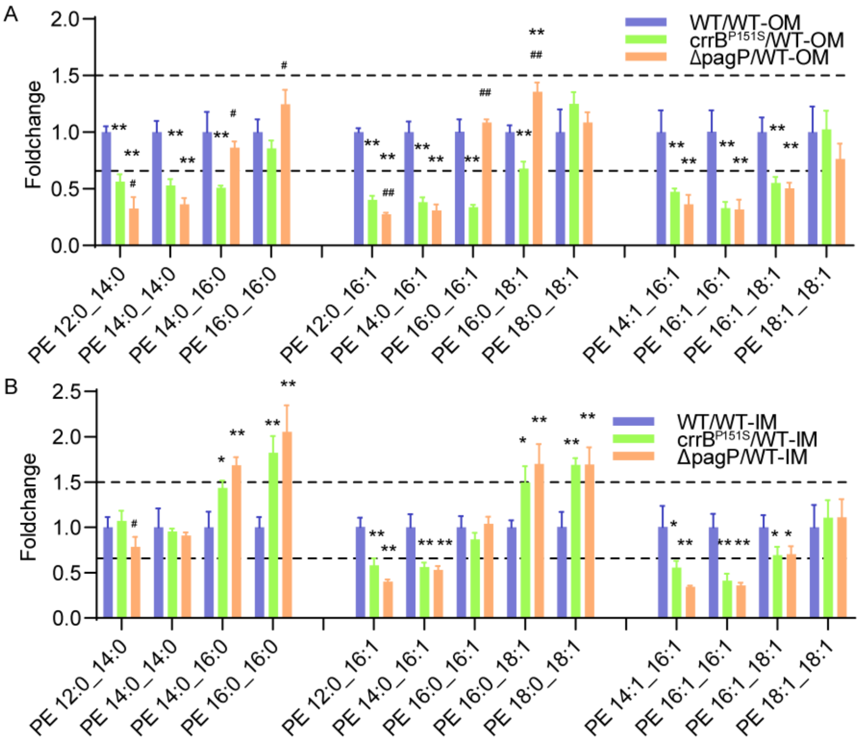


Fig. S3. The alterations of phosphatidylethanolamine contents within the outer and inner membrane. (A) Decreased OM PE contents with a palmitate chain in the *crrB* mutant were increased towards the level of the wild type, while that without a palmitate chain showed a negligible alteration after *pagP* knockout. (B) PagP inactivation had a minor impact on the PE content with the inner membrane in the *crrB* mutant. The experiment was repeated three times with four technical replicates each and the data was in Supplementary File 4. *(p-value<0.05) or **(p-value<0.01) indicates a significance of *crrB*$^{P151S}$ or *ΔpagP* compared to WT. #(p-value<0.05) or ##(p-value<0.01) shows a significance of *ΔpagP* in comparison to *crrB*$^{P151S}$.

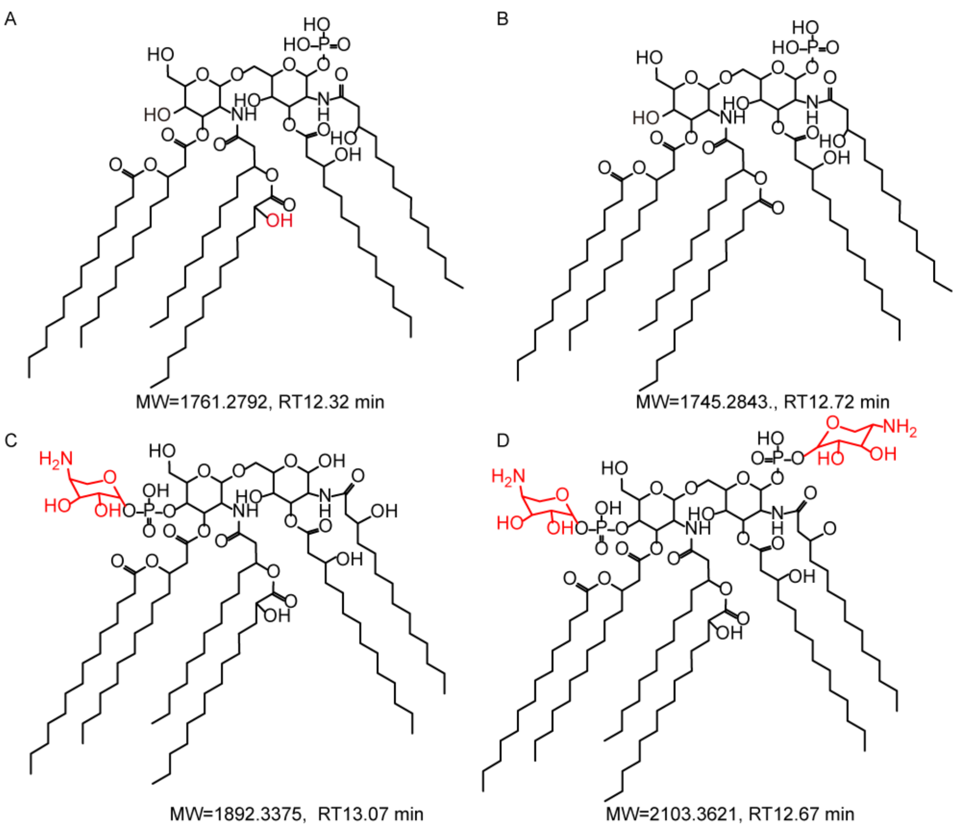


Fig. S4. The representative structures of lipid A correspond to the peaks at the retention time of 12.32 (A), 12.72 (B), 13.07 (C) and 12.67 min (D).

Table S1. Primers for gene knockout used in the study

| Primer | Sequence (5'to 3') | nucleotide |
|---|---|---|
| pagP-spacer-F2 | tagtTTTAATAAGTGGGAGCCCAT | pagP spacer for gene deletion |
| pagP-spacer-R2 | aaacATGGGCTCCCACTTATTAAA | pagP spacer for gene deletion |
| pagP-LF | ACCGACATTCACCATTACC | amplification of *pagP* for validation |
| pagP-LR | TGACCTTCAGCCAGAGTT | amplification of *pagP* for validation |
| pagP-template | CACAAGACCTTCCGCTTATCTCATTTATTGC TAAGACTTAGCGAACAACACTTTTCTATTAC CACTTTGATTTAAAAGCCACCTAAAATA | 90 nt *pagP* donor template for gene deletion |
| mlaC-spacer-F2 | tagtCGGTACGTCTTGACTTCCAG | *mlaC* spacer for gene deletion |
| mlaC-spacer-R2 | aaacCTGGAAGTCAAGACGTACCG | *mlaC* spacer for gene deletion |
| mlaC-LF | CTGGTGAACTGCCTGATTA | amplification of *mlaC* for validation |
| mlaC-LR | GTAGATGCTCTGCTGCTT | amplification of *mlaC* for validation |
| mlaC-template | ACGCCGGCAGCCGGCACGACGCATTAATTT CAGGAGAAATACGATTGAGCGGGCAGCTG AGCTGGACTAGCGAGGGCGAGACGCTGGC GC | 90 nt *mlaC* donor template for gene deletion |

Table S2. The fragmentation patterns of the identified lipids based on MRM on negative mode

| ID | Q1 | Q3 | CE | ID | Q1 | Q3 | CE |
|---|---|---|---|---|---|---|---|
| acyl-PG 42:0 | 875.638 | 227.202 | -50 | PG 34:1 | 747.518 | 281.249 | -40 |
| acyl-PG 44:0 | 903.67 | 227.202 | -50 | PG 36:1 | 775.549 | 281.249 | -40 |
| acyl-PG 46:0 | 931.701 | 255.233 | -50 | PG 30:2 | 689.44 | 253.217 | -40 |
| acyl-PG 48:0 | 959.732 | 255.233 | -50 | PG 32:2 | 717.471 | 253.217 | -40 |
| acyl-PG 50:0 | 987.763 | 255.233 | -50 | PG 34:2 | 745.503 | 253.217 | -40 |
| acyl-PG 42:1 | 873.623 | 253.217 | -50 | PG 36:2 | 773.534 | 281.249 | -40 |
| acyl-PG 44:1 | 901.654 | 253.217 | -50 | LPE 14:0 | 424.247 | 227.202 | -40 |
| acyl-PG 46:1 | 929.685 | 253.217 | -50 | LPE 14:1 | 422.231 | 225.186 | -40 |
| acyl-PG 48:1 | 957.717 | 255.233 | -50 | LPE 16:0 | 452.278 | 255.233 | -40 |
| acyl-PG 50:1 | 985.748 | 255.233 | -50 | LPE 16:1 | 450.263 | 253.217 | -40 |
| acyl-PG 52:1 | 1013.779 | 281.249 | -50 | LPE 18:0 | 480.31 | 283.264 | -40 |
| acyl-PG 44:2 | 899.638 | 253.217 | -50 | LPE 18:1 | 478.294 | 281.249 | -40 |
| acyl-PG 46:2 | 927.67 | 253.217 | -50 | PE 26:0 | 606.414 | 227.202 | -40 |
| acyl-PG 48:2 | 955.701 | 253.217 | -50 | PE 28:0 | 634.445 | 227.202 | -40 |
| acyl-PG 50:2 | 983.732 | 253.217 | -50 | PE 30:0 | 662.477 | 227.202 | -40 |
| acyl-PG 52:2 | 1011.763 | 281.249 | -50 | PE 32:0 | 690.508 | 255.233 | -40 |
| acyl-PG 54:2 | 1039.795 | 281.249 | -50 | PE 28:1 | 632.43 | 253.217 | -40 |
| acyl-PG 46:3 | 925.654 | 253.217 | -50 | PE 30:1 | 660.461 | 253.217 | -40 |
| acyl-PG 48:3 | 953.685 | 253.217 | -50 | PE 32:1 | 688.492 | 253.217 | -40 |
| acyl-PG 50:3 | 981.717 | 253.217 | -50 | PE 34:1 | 716.524 | 281.249 | -40 |
| acyl-PG 52:3 | 1009.748 | 281.249 | -50 | PE 36:1 | 744.555 | 281.249 | -40 |
| acyl-PG 54:3 | 1037.779 | 281.249 | -50 | PE 30:2 | 658.445 | 253.217 | -40 |
| LPG 16:0 | 483.273 | 255.233 | -50 | PE 32:2 | 686.477 | 253.217 | -40 |

| ID | Q1 | Q3 | CE | ID | Q1 | Q3 | CE |
|---|---|---|---|---|---|---|---|
| LPG 16:1 | 481.257 | 253.217 | -40 | PE 34:2 | 714.508 | 253.217 | -40 |
| LPG 18:1 | 509.288 | 281.249 | -40 | PE 36:2 | 742.539 | 281.249 | -40 |
| PG 30:0 | 693.471 | 227.202 | -40 | 16:0-d31-18:1 PE | 747.718 | 281.249 | -40 |
| PG 32:0 | 721.503 | 255.233 | -40 | 16:0-d31-18:1 PG | 778.713 | 281.249 | -40 |
| PG 28:1 | 663.424 | 253.217 | -40 | Palmitic aicd-d31 | 286.593 | 286.593 | -40 |
| PG 30:1 | 691.456 | 253.217 | -40 | 16:0 LPE-d9 | 461.335 | 264.289 | -40 |
| PG 32:1 | 719.487 | 253.217 | -40 | | | | |
